# The Ability of Near Infrared (NIR) Spectroscopy to Predict Functional Properties in Foods: Challenges and Opportunities

**DOI:** 10.3390/molecules26226981

**Published:** 2021-11-19

**Authors:** Daniel Cozzolino

**Affiliations:** Centre for Nutrition and Food Sciences, Queensland Alliance for Agriculture and Food Innovation (QAAFI), The University of Queensland, St. Lucia, Brisbane, QLD 4072, Australia; d.cozzolino@uq.edu.au

**Keywords:** NIR, spectroscopy, functionality, food, composition

## Abstract

Near infrared (NIR) spectroscopy is considered one of the main routine analytical methods used by the food industry. This technique is utilised to determine proximate chemical compositions (e.g., protein, dry matter, fat and fibre) of a wide range of food ingredients and products. Novel algorithms and new instrumentation are allowing the development of new applications of NIR spectroscopy in the field of food science and technology. Specifically, several studies have reported the use of NIR spectroscopy to evaluate or measure functional properties in both food ingredients and products in addition to their chemical composition. This mini-review highlights and discussed the applications, challenges and opportunities that NIR spectroscopy offers to target the quantification and measurement of food functionality in dairy and cereals.

## 1. Introduction

A functional food is a term utilized to describe food or food ingredients that can provide health benefits in addition to its basic nutrition [1]. Functional foods include ingredients and any edible product in which the concentration of one or several bioactive compounds or phytonutrients such as vitamins, antioxidants and micronutrients are present [1]. It is well known that fruits and vegetables are the main sources of dietary phytonutrients, especially antioxidants (e.g., polyphenols and flavonoids, vitamins, glucosinolates, carotenoids, etc.). Several epidemiological studies and clinical trials have suggested and demonstrated that diets rich in fruits and vegetables can reduce the risk of chronic diseases [1]. Furthermore, frequent intake of phytonutrients could potentially prevent cancer, cardiovascular diseases, diabetes, osteoporosis and age-related disorders such as dementia [2,3,4]. Therefore, most of these phytochemicals are measured and quantified by using a wide range of analytical methods and techniques including high performance liquid chromatography (HPLC), liquid chromatography (LC), mass spectrometry (MS) and vibrational spectroscopy (e.g., near (NIR) and mid (MIR) infrared, Raman, UV and visible spectroscopy) [2,3,4].

**Figure 1:**
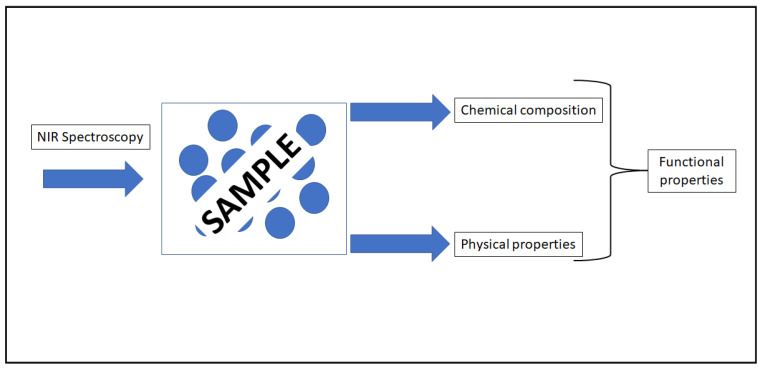
The utilization of near infrared spectroscopy to measure chemical and physical properties in foods.

In addition to the natural presence of these bioactive compounds in foods, processing (e.g., extrusion, drying and fermentation) also plays an important role in determining or altering not only the chemical composition and concentration of these bioactive compounds in food but also its functional properties (see Figure 1). This is of particular importance in industries such as the dairy and cereals where it is well known that changes in particle size or the effect of temperature during processing, to mention a few, can have an impact on the functional properties of the raw ingredient and final food product.

This mini-review highlights and discussed the applications, challenges, and opportunities that near infrared (NIR) spectroscopy offers to target the quantification and measurement of food functionality in dairy (e.g., milk powder) and cereals (e.g., wheat, rice and bakery products).

## 2. Near Infrared Spectroscopy

Near infrared spectroscopy deals with the interaction of matter and light in the near infrared region of the electromagnetic spectrum between 750 and 2500 nm [5,6,7,8]. When the infrared light interacts with the molecules present in a sample, the bond of these molecules vibrates at different frequencies depending on the type of bond (e.g., energy bonding) [5,6,7,8]. In the NIR region, the C–H, N–H and OH vibration bonds are the most prevalent, determining the shape of the spectra of a given sample. Consequently, the resultant spectrum is characterized by the overlap of these bonds in broad bands [5,6,7,8]. Although most of the applications of NIR spectroscopy deal with the measurement and/or quantification of the proximate composition (e.g., protein) of a sample, the NIR spectrum also reflects the physical properties or characteristics of the sample [5,6,7,8]. This is a unique characteristic of NIR spectroscopy that differentiates this technique from other instrumental techniques. Therefore, the NIR spectra of samples can provide information not only about the chemical composition of food but also about its functionality [5,6,7,8].

The implementation of NIR spectroscopy into a robust analytical method requires the utilization of computational and data analytic tools [9,10,11,12,13]. These tools are needed to extract the relevant information from the data collected by NIR spectroscopy during the analysis and to develop mathematical models that can be used to better understand the system analyzed [9,10,11,12,13]. Most of these data analytical methods are based on a wide range of algorithms and statistical techniques (e.g., multiple linear regression, partial least squares regression and principal component analysis) [9,10,11,12,13]. Overall, the routine use of NIR spectroscopy requires, in most cases, the development of a calibration. The calibration describes the relationships between the spectra and the reference data, and it is expressed as a mathematical model [9,10,11,12,13]. The calibration is evaluated by its capability to predict new samples and by evaluating how good it is in relation to the reference method used [9,10,11,12,13]. Calibrations are often needed during the implementation of NIR spectroscopy, as this technique is a low-selective technique and, unlike the mid infrared (MIR) spectra, captures only the overtones and combination tones of vibrations derived from the functional bonds such as CH, OH, NH and SH [9,10,11,12,13]. During the analysis of samples using NIR spectroscopy, overtones appear as highly overlapped peaks and require the extensive use of chemometrics in order to process and extract the signal related to the property of interest [9,10,11,12,13]. Once the NIR spectrometer is calibrated, it can be deployed for routine use but requires extensive testing to confirm their predictive ability or to monitor any changes associated with the failure of the sensor, light source, electronics, etc. [9,10,11,12,13]. In the following section the utilization of NIR spectroscopy to measure and quantify different functional properties in foods as reported by other authors is described.

## 3. Examples on the Use of Near Infrared Spectroscopy to Measure Food Functionality

### 3.1. Dairy Products

The physicochemical characteristics of food ingredients and products were assessed using different visible (VIS) and NIR spectroscopy instruments (e.g., portable, benchtop and hyperspectral imaging) as reported by different authors [14,15,16,17,18,19,20]. The utilization of NIR spectroscopy to evaluate the functional properties of dairy products was evaluated in a recent study [21]. The variability in the dataset was obtained by collecting samples from different batches, brands, types and length of storage [21]. The results of this study reported moderate to good performance on the quantification and measurement of parameters such as tapped density, insolubility index, surface free fat, moisture content and bulk density where the coefficient of determination ranged from 0.65 to 0.88 [21]. These authors analyzed the different models by using the variable of importance in projection and selectivity ratios, showing the uniqueness of each prediction model for the different physicochemical attributes measured in dairy powder samples [21].

The utilization of NIR spectroscopy to predict fine particle size fraction, dispersibility and bulk density of various milk powder samples was reported [22]. Partial least squares (PLS) regression was used to develop predictive models by using the NIR spectra of milk powder samples and the physical and functional properties defined above [22]. The authors of this study concluded that the PLS models predicted milk powder properties with an accuracy of 88–90 percent [22].

The size of colloidal particles in food products has considerable impacts on the physicochemical, functional and sensory characteristics of a dairy product [23]. The utilization of visible (VIS) and NIR spectroscopy was used to monitor and quantify fat globule size in milk samples [23]. In this study, the variability in fat globule size distribution was created using ultrasonic homogenization of raw milk [23]. The authors reported that reductions in fat globule size resulted in a higher wavelength dependency of both the VIS and NIR spectra bulk scattering coefficient and the scattering anisotropy factor [23]. The anisotropy factor and the bulk scattering coefficients for wavelengths above 600 nm were reduced and dominated by Rayleigh scattering [23]. In this study, the bulk scattering properties could be estimated (R^2^ ≥ 0.90) by measuring particle size distribution using an algorithm based on the Mie solution [23]. The authors suggested that the inversion of this model can be used to estimate particle size distributions by using VIS and NIR spectroscopy [23].

The effect of physicochemical factors, as well as the utilization of skim milk powder on milk rennet-coagulation, was evaluated by using NIR spectroscopy and multivariate curve resolution alternating least squares (MCR-ALS) [24]. Coagulum formation has been evaluated by using reference methods as well as by the utilization of NIR spectroscopy analyzing unaltered reconstituted milk samples, pasteurized samples, samples with calcium chloride addition and samples of reconstituted milk mixed with fresh milk [24]. The calibration models were able to monitor changes during processing, explaining more than 99.9% of variance in the dataset [24]. The authors concluded that the NIR models were able to monitor a diverse range of coagulation conditions, demonstrating the suitability of this technique for evaluating the rennet-induced coagulation of reconstituted milk [24].

The contents of common dietary fatty acids (FAs) and fat content in liquid milk were evaluated by NIR spectroscopy with the aim of improving cow feed management practices [25]. In addition, these authors have used aquaphotomics to better interpret the interactions between water molecular structure and FAs as a tool to better understand liquid milk functionality [25]. In this study, milk samples (n > 250) were collected from different cows for 14 weeks where FA content was measured by using gas chromatography (GC) [25]. The utilization of aquaphotomics, applied to NIR spectroscopy, was used to quantify these fatty acids in the 1300–1850 nm region. This study showed the potential of NIR spectroscopy to quantify dietary FAs [25]. The calibration models were externally validated, and good prediction statistics were obtained (R^2^ > 0.75, RPD >1.5) for caproic and caprylic acids and acceptable for capric, lauric, myristic, myristic-oleic, palmitoleic and oleic acid [25]. The analysis and interpretation of the most important NIR wavelengths that contributed to developing the regression models indicated that the contribution of water bands associated with protonated and hydration water might be explained by the interaction of water and fatty acids. The authors of this study concluded that these interactions are relevant for their self-organization into assemblies with different morphologies in the food matrix [25].

### 3.2. Cereals and Starchy Foods

The term quality in cereals (e.g., wheat and barley) is complex and depends upon the different characteristics of the grain, environment, and end use [26,27]. Endosperm texture (grain hardness), protein content and gluten strength are considered the main drivers of wheat quality [26,27]. For example, endosperm texture in wheat is the single most important and defining quality characteristic, as it facilitates wheat classification and affects milling, baking and end-use quality [26,27]. Various techniques used for grain hardness measurement are classified into diverse groups according to grinding, crushing and abrasion [26,27]. Different methods have been used to measure texture, and they include particle size indicator (PSI), NIR hardness, single kernel characterization system (SKCS), pearling index, sodium dodecyl-sulfate polyacrylamide gel electrophoresis (SDS-PAGE) and polymerase chain reaction (PCR) markers [26,27].

It is well known that wheat gluten plays an important role in the bread and milling food industry [28], particularly in baking to help standardize dough properties and improve bread volume [28]. However, a comprehensive characterization of a wide variety of gluten samples is not available where this would be necessary to relate compositional characteristics to the production process [28]. In this study, the researchers measured crude protein, starch, lipids and ash content, oil and water absorption capacity, particle size distribution, gluten protein composition and the NIR spectra of gluten samples from different suppliers [28]. The authors have utilized principal component analysis (PCA) to analyze the trend and sources of variability in the dataset from different compositions and producers [28]. It was observed that the composition of vital gluten samples from the same manufacturer was similar, and the score plot showed a cluster formation for samples from three suppliers; the variability over all samples was comparatively low [28]. The samples from the other suppliers were too similar altogether, so it was hardly possible to identify clear differences that were also related to functionality [28].

Changes in physicochemical properties during rice storage at high temperatures was reported [29]. In this study, paddy rice cultivar samples having low amylose (9–11%) and high amylose content (23–25%) were stored at 39.2 °C and 43.1% relative humidity up to 31 weeks [29]. The authors have reported changes in moisture content and other parameters associated with cooking quality, texture and pasting properties during the first four weeks of storage [29]. Predictive models based on NIR spectroscopy were developed for the prediction of minimum cooking time, adhesiveness, pasting temperature, peak viscosity and breakdown of rice samples (R^2^ of calibration ≥ 0.81; R^2^ of validation ≥ 0.87) [29].

The use of single-kernel NIR (SKNIR) was used to analyze the effects of fractioning heterogeneous bulk wheat [30]. This study showed that sorting wheat into three functionality fractions was performed on low quality lots from an organic field experiment from two growth years and two locations [30]. The sorted lots were mixtures that were originally diversified by three different preceding catch crops, resulting in 12 fractions, in addition to the two original wheat lots that were characterized by 20 standard quality variables of grains and flours [30]. The data were analyzed by using PCA and analysis of variance (ANOVA) [30]. Within each harvest and location/cultivar, SKNIR fractionation had significant positive effects on bulk grain density, protein, wet gluten content, Zeleny sedimentation volume, farinograph water absorption, farinograph softening, failing number, gelatinization temperature and hardness index [30]. This study indicated that the direct use of the NIR fingerprint region can be used in sorting samples without calibration to a univariate reference, which showed that the resulting fractions were based on the major variance in the entire physicochemical quality trait within each lot as expressed by NIR [30]. This study has demonstrated that unsupervised approaches can be considered as a powerful tool for sorting samples according to complex functionality traits, thus increasing overall quality, applicability, and value of the sorted crop [30].

Two-dimensional (2D) correlation analysis was used to characterize NIR wheat [31]. Before 2D analysis, the NIR spectra possessing neighboring protein reference values were averaged, and then the new spectral set was subjected to principal component analysis for cluster classification [31]. The same approach was repeated for the determination of sodium dodecyl sulphate sedimentation (SDSS) volume [31]. Both synchronous and asynchronous 2D correlation spectra enhanced spectral resolution and provided information about protein content and SDSS index-dependent intensity changes not readily accessible from one-dimensional NIR spectra of wheat [31]. The results showed differences between the protein content and SDSS index induced NIR spectral response [31]. The analysis of the NIR spectra showed several unique protein wavelengths around 1980 nm, 2040 nm, 2200 nm, 2260 nm and 2350 nm in high protein and SDSS wheat [31]. The authors of this study indicated that at least one of these wavelengths could be associated with gluten protein (a complex formed primarily from gliadin and glutenin) that influences the end-use quality of wheat flour [31].

Commercial bakeries need rapid and simple means to measure the dough-handling and bread-making functionalities of wheat flour during processing [32]. The ability of NIR spectroscopy was explored to measure both dough and bread making properties [32]. Samples were sourced from flour from both hard red spring and hard red winter wheats. Samples were analyzed by water absorption, dough mixing time, dough mixing tolerance, loaf height, internal grain appearance and overall bake score [32]. The authors indicated that the ability of NIR spectroscopy to measure protein, starch damage and positive relationships between the two and water absorption probably made it successful for modelling water absorption [32]. Models for the remaining five indices were less accurate due to the complexity of interactions between protein, starch and lipids and inadequate instrument sensitivity [32].

The ability of NIR spectroscopy was evaluated in monitoring heat-treatment effects in the case of wheat milling fractions and changes in the quality in these fractions [33]. The heat treatment processes are being applied to increase the shelf-life properties of cereal products or to change the physical/rheological properties [33]. Wheat products and fractions have been produced under industrial conditions where the following analyzed characteristics were applied, such as Hungarian wheat fraction (WF), Hungarian cake flour (CF) and aleurone-rich wheat flour (ARF) [33]. Changes in the main chemical components (such as starch and protein) were analyzed with dispersive spectrophotometers by using visible and NIR regions of the electromagnetic radiation with regards to the heat treatments [33]. A close correlation has been established between the data of spectroscopic measurement techniques processed by various chemometric methods (e.g., principal component analysis and cluster analysis) and the types of treatments that were used [33]. Not only differences caused by the milling technology and the heat treatment settings have been clearly observed but also differences between dry-thermal and hydrothermal treatments [33]. During this task, it became obvious that the NIR methods can detect the deviation in parameters of the heat treatments [33].

Farinograph tests are used to measure the functional properties as well as the quality of wheat flour [34]. This test was recognized to be time consuming and labor intensive [34]. Some studies have shown that NIR spectroscopy showed a limited ability to predict farinograph parameters; therefore, the combinations of NIR and MIR spectroscopy were evaluated to predict wheat flour farinograph quality properties (water absorption, dough development time, dough stability and degree of softening) [34]. Calibrations were developed by using PLS regression and NIR, MIR and the fused spectra [34]. Data fusion strategies were applied by the authors to take advantage of the synergistic effect of information obtained from MIR and NIR spectroscopy [34]. Low-level data fusion models showed inferior performance compared to the corresponding MIR and NIR models, whereas mid-level data fusion models combined with a forward interval variable selection algorithm were validated to show good performance. The authors indicated that the fusion of the previously selected variables from MIR and NIR spectra improved the prediction accuracy of farinograph parameters, which indicates the superiority of the forward interval variable selection algorithm that will be helpful the cereal and baking industries [34].

The ability of NIR spectroscopy to predict chemical composition (protein, moisture, fat, ash and total dietary fibre) and functional properties (100 seed weight, hydration capacity, hull percentage, dehulling efficiency, colour and cooking quality) in field peas and chickpeas was reported [35]. The accuracy of the NIR calibration models was generally better on ground than on entire samples for chemical composition but better on whole than ground samples for physical or functional properties [35]. For most properties measured, the accuracy of NIR calibrations was considered either satisfactory or promising, but successful implementation in a breeding programme will depend on further evaluation using independent samples, broadening the sample populations or improvements in the reference methods [35].

Recent reviews and reports have also highlighted the ability of NIR spectroscopy to predict different parameters associated with starch physicochemical characteristics, most of them associated with functional properties in cereals and other starchy foods [36,37,38,39].

## 4. Challenges and Opportunities

It is not surprising that NIR spectroscopy is considered one of the most utilised routine analytical methods by the food industry. The advantage of this technique is based on its ability to determine proximate composition in foods such as protein, moisture or dry matter, fat and starch via a technique that is non-destructive, rapid and green (e.g., no reagents used). As demonstrated and discussed in this short review, several studies highlighted the ability of NIR spectroscopy to predict or measure properties that are associated with the functionality of food ingredients and products such as dairy and cereals. This ability is the result of the well-known but little explored properties of NIR spectroscopy that are not only capable of measuring and or quantifying the proximate composition of a sample but also reflects the physical properties and/or functional characteristics of the same sample. However, the prediction or quantification of these functional properties in foods by NIR spectroscopy is beyond the sole prediction of a given parameter. The analysis and interpretation of the NIR spectrum is providing more information than can be used in revealing such properties that are intimately associated with functionality.

Although the utilization of NIR spectroscopy is allowing for the development and implementation of advanced food management decision systems, the translation of NIR spectroscopy across the food industry as tool to monitor functionality is still not well established. The information available about the prediction or measurement of both chemical composition and functional properties by NIR spectroscopy will provide new means to improve and incorporate decisions management tools when processing foods. Furthermore, the current development in hardware and software will result in an increase in NIR capability to be used during the evaluation and monitoring of food quality along the entire food supply chain and value chains.

Despite its huge potential and the use of NIR spectroscopy to meet online inspection and quality prerequisites imposed by the food industry, the measurement of functional properties will require further developments in R&D as well as in training. The development of applications of NIR spectroscopy often requires the creation of calibration models that must include different environmental conditions, samples from different and diverse origin and appropriate spectral pre-processing. However, the importance of this critical step is somehow ignored or underestimated. Additionally, the current global demand for fully nutritious, sustainable and safe foods is on top of the agenda for the modern food industry and, more importantly, for the consumer; this is challenged by several overarching issues, including increasing complexity in the food supply chains, the effect of climate change (e.g., composition of raw materials and ingredients), growing ageing population, food security issues (e.g., fraud and waste) and the continuous changes in consumers patterns or choices for food nutritious and safe foods.

The integration and utilization of NIR spectroscopy as a routine method by the food industry has allowed for a surge in our ability to acquire data in a fast and efficient manner; therefore, the capability of using such information in different ways is available to us, for example, monitoring the entire supply and value chain from processing, transport, and storage of foods. However, how information can be incorporated or utilized by the food industry in the so-called management decision or expert systems are still lacking. In spite of its enormous advantage, the utilization of NIR spectroscopy in online or processing monitoring will bring novel options and tools for determining the composition of foods. The utilization of NIR spectroscopy with other sensing technologies, data analytics and the Internet of Things will add a new dimension to better understand food systems.

However, users need to have a different approach in the interpretation of NIR models developed to predict food functionality, as the interpretation of functionality will determine a different level of complexity. The user needs to move away from a single number that explains what has already happened to a more dynamic system that will provide information about the changes or trends in the system as a function of its complexity. This new methodology will require the use of new tools (e.g., aquaphotonics) as well as to embrace multidisciplinary approaches to interpret NIR spectra and the calibrations obtained to predict food functionality.

## Data Availability

Not applicable.
